# Zeolites with Continuously Tuneable Porosity**

**DOI:** 10.1002/anie.201407676

**Published:** 2014-10-05

**Authors:** Paul S Wheatley, Pavla Chlubná-Eliášová, Heather Greer, Wuzong Zhou, Valerie R Seymour, Daniel M Dawson, Sharon E Ashbrook, Ana B Pinar, Lynne B McCusker, Maksym Opanasenko, Jiří Čejka, Russell E Morris

**Affiliations:** EaStCHEM School of Chemistry, University of St. AndrewsSt. Andrews KY16 9ST (UK); Department of Synthesis and Catalysis, J. Heyrovský Institute of Physical Chemistry, Academy of Sciences of Czech Republicv.v.i. Dolejškova 3, 18223 Prague (Czech Republic); Laboratory of Crystallography, ETH ZürichWolfgang-Pauli-Strasse 10, CH-8093 Zürich (Switzerland)

**Keywords:** ADOR, germanosilicates, porosity, zeolites

## Abstract

Zeolites are important materials whose utility in industry depends on the nature of their porous structure. Control over microporosity is therefore a vitally important target. Unfortunately, traditional methods for controlling porosity, in particular the use of organic structure-directing agents, are relatively coarse and provide almost no opportunity to tune the porosity as required. Here we show how zeolites with a continuously tuneable surface area and micropore volume over a wide range can be prepared. This means that a particular surface area or micropore volume can be precisely tuned. The range of porosity we can target covers the whole range of useful zeolite porosity: from small pores consisting of 8-rings all the way to extra-large pores consisting of 14-rings.

Zeolites are some of the most important solids in modern technology,[1] and the search for new types of zeolites and new methods for their preparation remains at the forefront of research.[2–9] The crucial feature of zeolites is their porosity, which defines their utility by governing the size and accessibility of the internal surface area where most of the important chemistry occurs. This in turn determines the chemical activity and selectivity of the materials. A major goal in zeolite science (and indeed in all science dealing with porous materials) is to control the porosity. Traditional zeolite synthesis only allows coarse stepwise tuning of the porosity based on the use of organic structure-directing agents (SDAs) or templates.[10,11] The porosity of a different type of porous material, called metal–organic frameworks, is normally regarded to be much more tuneable through a mechanism called reticular synthesis.[12] However, even in this process the porosities that can be obtained are not continuous, as they are limited by discrete changes in the length of the organic linkers that determine the pore sizes.

Here we describe a template-free mechanism by which the internal surface area and micropore volume of zeolites can be continuously tuned over a wide range. This is achieved by controlling the rates of two competing processes, silica de-intercalation and silica reorganization, by altering the conditions used to accomplish the hydrolysis and reassembly of a hydrolytically unstable parent zeolite. We report the structures of two new zeolites in this process, which we call IPC-6 and IPC-7.

The assembly–disassembly–organization–reassembly (ADOR) mechanism[13–16] is a new method of zeolite manipulation, in which the selective disassembly of a pre-prepared parent zeolite followed by reassembly can lead to new topologies. The key feature of the parent zeolite is the presence of a hydrolytically sensitive dopant element, especially Ge,[7–19] incorporated within the framework at a specific site (a double-four-ring (D4R) unit), which allows the chemically selective removal of the units containing the dopant.[15,16] ADOR has allowed the preparation of two new zeolite materials, IPC-2 and IPC-4.[13] IPC-2 has been given the IZA code **PCR**, while IPC-2 has the same connectivity (but different symmetry) to materials with the **OKO** code.[20] IPC-4 is a small/medium-pore zeolite that contains rings made of 8- and 10-tetrahedral units, while IPC-2 is a medium/large-pore material containing 10- and 12-rings.

IPC-4 is prepared through hydrolysis of Ge-UTL under neutral to slightly acidic conditions to form a layered intermediate (IPC-1P) followed by calcination at high temperature, sometimes in the presence of an organizing agent.[13] Zeolites with the **OKO** topology can be prepared by two methods, either directly through hydrolysis at very high acidity (e.g. 12 m HCl), the so-called inverse sigma transformation route, to give a material called COK-14,[21] or by the intercalation of silica species into IPC-1P, prepared by hydrolysis at low acidity, to give IPC-2.[13,16] IPC-2 and COK-14 differ in their structural details (e.g. symmetry and composition), but share the same framework connectivity. The difference between the **OKO** (IPC-2) and **PCR** (IPC-4) topologies is that IPC-2 contains silica subunits (single-four-ring (S4R) unit) between **UTL**-like layers, whereas IPC-4 has no S4R units. At first this seems counterintuitive; one might imagine that highly acidic solutions should provide more effective hydrolysis conditions than near-neutral ones, but the reverse seems to be the case: hydrolysis under near-neutral conditions removes all the D4R units from between the **UTL** layers, while hydrolysis using 12 m HCl removes only half the D4R units.

Mechanistic studies on the ADOR process at different acidities showed that its outcome depends on two competing processes: silica reorganization and silica de-intercalation (Figure 1). We then targeted new zeolite frameworks that formed when the rates of silica reorganization and silica de-intercalation were varied. The key feature of the study is that by controlling the rates of the processes, the porosity of the final material is tuneable, and that this porosity control covers the whole range of useful zeolite porosities, from 8-ring materials with small pores all the way to 14-ring materials with extra-large pores.

**Figure 1:**
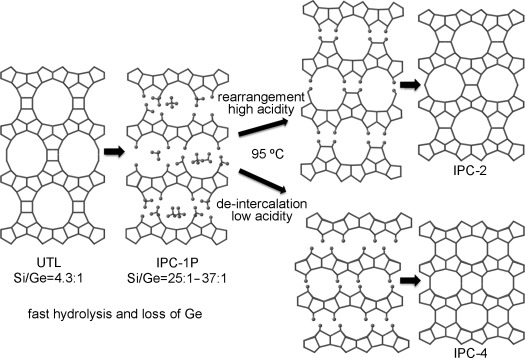
Acidity-dependent two-step hydrolysis mechanism of the ADOR process.

Ge-UTL samples (Si/Ge ratios 4.3:1 and 6.0:1) were prepared using reported approaches.[13] The synthetic protocol used to explore the mechanism is given in the Supporting Information, and essentially involves careful hydrolysis of the parent zeolite under different conditions for different lengths of time.

The solids recovered after hydrolysis for five minutes at room temperature showed little variation with the conditions (see the Supporting Information, Figure S1). The only signals that changed greatly were those associated with the interlayer spacing, while those from the layers themselves were almost unchanged. The ratio of Si/Ge remaining within the recovered solids increased from 4.4:1 to between 25:1 and 37:1, showing no particular pattern with the change in acidity of the solution. In solid-state NMR analyses (see the Supporting Information), all the samples hydrolyzed for five minutes at room temperature showed similar spectra, containing Q4-, Q3-, and Q2-type Si atoms in an average ratio of 74.6:24.8:0.6. These results indicate that the initial hydrolysis is relatively fast and does not depend greatly on the acidity of the hydrolysis solution.

Upon heating at 95 °C, the concentration of the acid had a significant effect on the outcome of the reaction. Using 12 m HCl, some of the samples that were hydrolyzed for one or two hours showed a reduction in crystallinity and sometimes an amorphous feature in the powder X-ray diffraction (PXRD) data. After 8, 16, and 24 hours, the crystallinity of the samples returned, but the main reflection in the PXRD showed a change to smaller scattering angles (2*θ*≈7.7°). This result is consistent with a rearrangement process in which Si atoms are removed from the layers and reposition between the layers. Calcination of this sample generally leads to IPC-2. It is known that highly acidic conditions promote the making/breaking of silica bonds and the results seen in these experiments fit these observations.[22] The final Si/Ge ratio in the samples is always higher than 200:1, thus indicating that more than 99 % of the Ge has been removed during this process.

In contrast, using neutral or lightly acidic conditions, the main peak in the PXRD shifted to slightly higher scattering angles (2*θ*≈8.5°). There was a slight decrease in the interlayer spacing, and EDX analysis showed that further Ge atoms had been de-intercalated from the solid (the final Si/Ge ratio was in the range 80–100:1 for all these samples).

It is clear that there are two competing processes at work (Figure 1). High acidity favors the rearrangement of silica from the layers to the interlayer sites, while neutral and only very mildly acidic conditions favor de-intercalation of any remaining species from between the layers. An important question to ask is whether there are conditions under which the two processes are equally likely, and whether this would produce a different outcome. We therefore varied the acidity of the solution (from neutral to an [H^+^]=12 m), performed an initial hydrolysis at room temperature, heated at 95 °C for 17 h, and finally calcinated the samples. All these reactions used Ge-UTL with a Si/Ge ratio of 6.0:1. Above an [H^+^] of approximately 8 m and under the same conditions described in reference [21], all samples produced the COK-14 variant of the **OKO** topology. Under neutral and 0.01 m conditions, IPC-4 was formed, but at [H^+^] between 0.5 m and 6 m, the 200 reflection, which is indicative of the interlayer spacing in the materials, shows a steady shift to a decreasing scattering angle (i.e. increasing interlayer spacing) with increasing acidity (Figure 2 a). The reflections in the XRD data corresponding to the **UTL**-like features, which are present in both IPC-2 and IPC-4, remain broadly unchanged. At [H^+^] between 0.1 m and 3 m, the change in interlayer distance (as measured by *d*_200_) is proportional to the [H^+^] used in the hydrolysis process (Figure 2 b).

**Figure 2:**
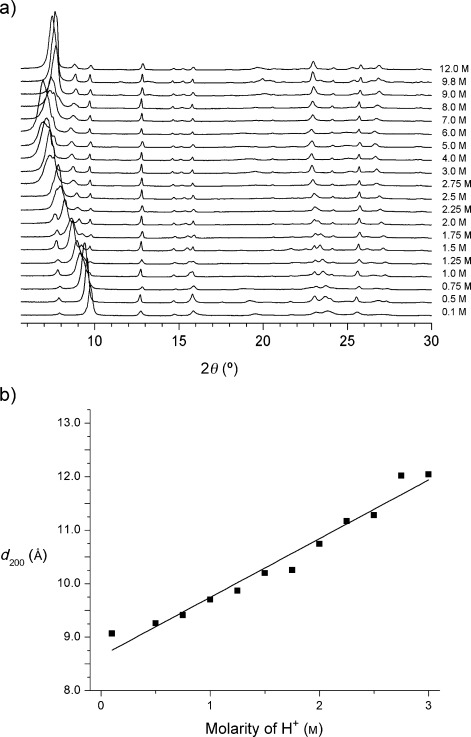
Dependence of powder X-ray diffraction patterns (a) and interlayer d-spacing (b) on the acidity of the hydrolysis solution. The straight line in (b) is fitted to the equation *d*_200_=[H^+^]+8.7. A slightly better fit can be achieved using a curved line, but the differences are small.

Under conditions with [H^+^] greater than 4 m, a more complex relationship (see the Supporting Information) with a maximum interlayer spacing reached at 5 m could be observed. Transmission electron microscopy (TEM) showed that the materials prepared at [H^+^] between 4 m and 8 m were less ordered and more defective than those prepared at [H^+^] below 4 m (see the Supporting Information, Figure S8).

A particularly important challenge in the research of zeolite materials is the tuneability of the internal surface area or pore volume. As IPC-2 connections lead to larger pores (12×10 rings) than IPC-4 connections (10×8 rings), increasing the acidity of the hydrolysis solution is a means to directly control the total porosity of the zeolite, as measured either by surface area or pore volume (Figure 3). There is a clear linear relationship between both the BET surface area and the micropore volume when plotted against [H^+^] up to 3 m, and a different linear relationship at [H^+^] between 3 m and 6 m. This means that the surface area of the resultant solids can be tuned over a range of surface areas (from around 150 to 590 m^2^ g^−1^) or micropore volumes (from about 0.06 to 0.22 cm^3^ g^−1^). This monotonic control over the pore volume and surface area is not possible using traditional synthetic approaches to zeolites and marks out the described de-intercalation/reorganization process as a particularly notable advance. The porosity reaches a maximum of 590 m^2^ g^−1^ at [H^+^]=5 m with a micropore volume of 0.22 cm^3^ g^−1^. The surface area then decreases to about 480 m^2^ g^−1^ at [H^+^]=9 m, and under conditions that produce COK-14, the surface area is invariant with [H^+^].

**Figure 3:**
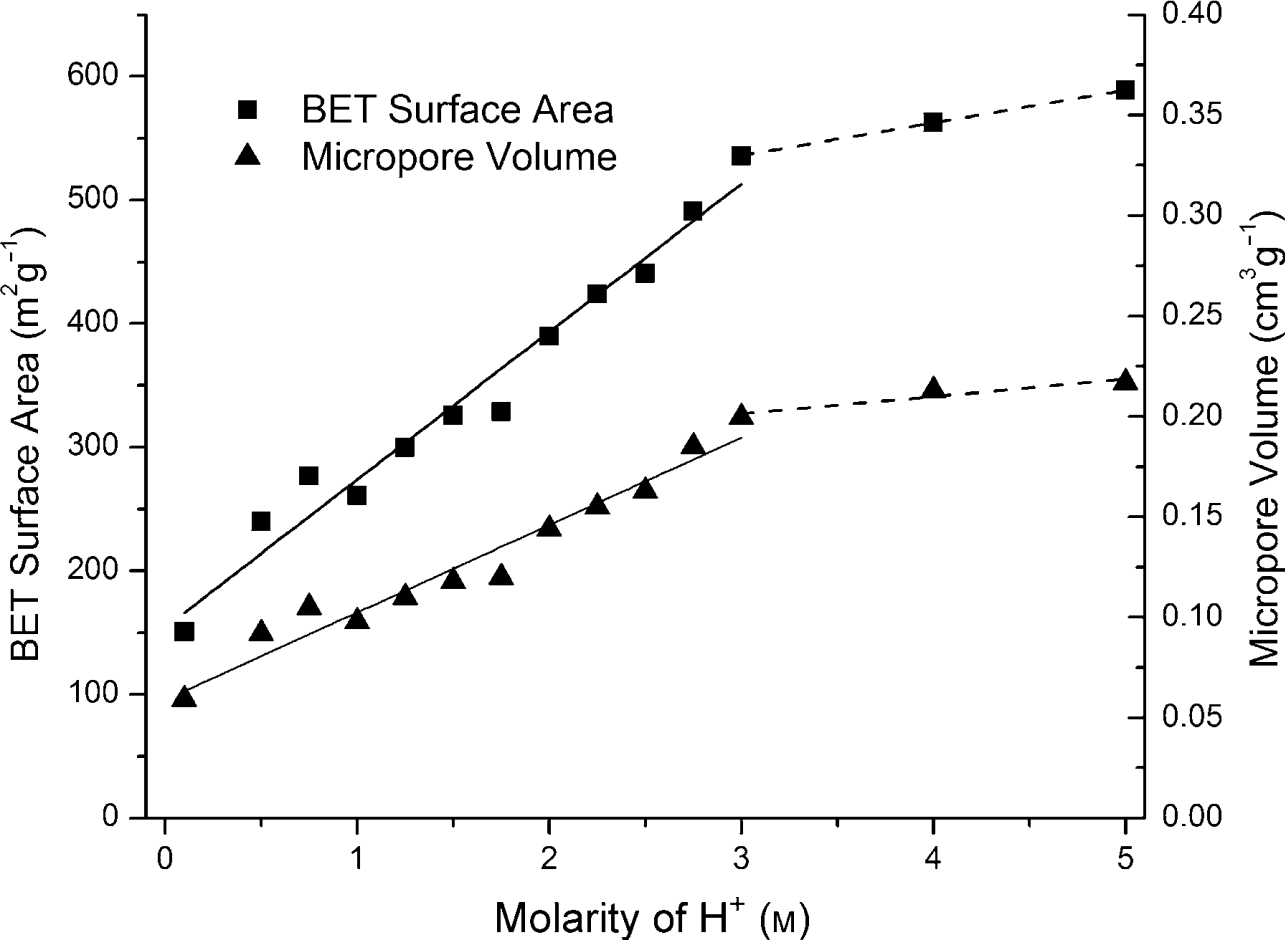
Relationships between BET surface area (left-hand axis) and micropore volume (right-hand axis) under hydrolysis conditions, showing how porosity is continuously tuneable

The changes in d-spacing, surface area, and micropore volume can all be explained by a staged de-intercalation/reorganization mechanisms, in which only a certain fraction of layers undergo the de-intercalation process and the rest undergo the rearrangement. The final materials will therefore contain different proportions of “IPC-2” connections (single-four-ring units between the layers) and “IPC-4” connections (only oxygen atoms between the layers), depending on the degree of staging. This is confirmed by the interlayer spacings measured by TEM. Above [H^+^]=3 m, a similar process explains the further increased porosity of IPC-7, but this time the rearrangement under higher acidity leads to some UTL-like connections consisting of double-four-ring units. Again this is confirmed by the TEM measurements that show interlayer spacings of both 1.4 nm (D4R spacers) and 1.1 nm (S4R spacers) present in the same crystals (see the Supporting Information, Figure S9).

To study the structure of IPC-6, we collected synchrotron PXRD data on the sample prepared at [H^+^]=1.5 m to improve the resolution (see the Supporting Information). The indexing of this pattern gave a C-centered unit cell, *a*=21.94, *b*=13.89, *c*=12.30 Å, and *β*=112°, with a *d*_200_ spacing midway between the IPC-2 and IPC-4 structures. There is also evidence of a broadening of the *h*00 reflections (i.e. along the stacking direction).

The model for this material is built as shown in Figure 4 a, with 50 % of the material undergoing the de-intercalation process to form IPC-4 connections and 50 % undergoing the rearrangement to form IPC-2 connections. This is equivalent to a stage-2-type de-intercalated material, in which each unit cell contains one of each type of connection, and is somewhat reminiscent (except of course in reverse) of staged intercalation of layered materials such as graphite[23,24] and some clays.[25]

**Figure 4:**
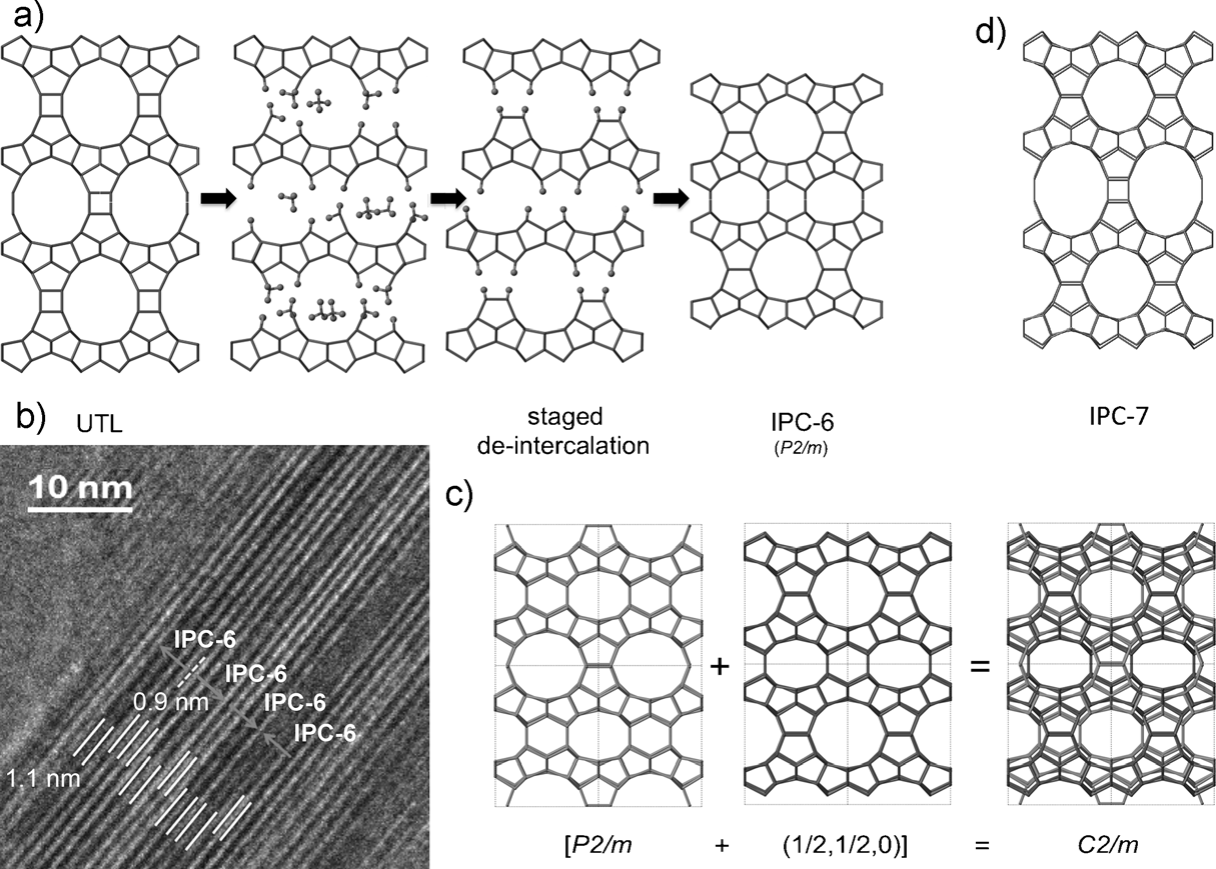
Staged structure of IPC-6. a) Formation of IPC-6 based on a staged de-intercalation mechanism. b) Representative TEM image showing the two different lattice fringe spacings (1.1 nm and 0.9 nm) and how two different settings of the IPC-6 unit cell can be used to describe the overall structure of the particles. c) Two orientations of IPC-6 give an average structure that has the same symmetry as the PXRD pattern. d) Structure of IPC-7.

However, the symmetry of this model is *P*2*m*, which does not fit with the *C*-centering observed in the PXRD pattern. TEM of the IPC-6 material was subsequently completed and showed clearly the two different layer spacings present in the material, with the expected lattice fringe separations of 1.1 nm and 0.9 nm for the IPC-2 and IPC-4 connections, respectively (Figure 4 b). The TEM image also explains the symmetry of the PXRD patterns, as the material is clearly composed of two different settings of the IPC-6 unit cell (two origins are possible). The TEM image also shows that IPC-6 is not an intergrowth structure of IPC-2 and IPC-4. The average structure is a combination of the two settings, and this combination is *C*-centered (Figure 4 c). Rietveld refinement of this model against the synchrotron X-ray pattern proceeded successfully and indicated that the model is consistent with the PXRD data, as well as with the TEM and porosity data.

A similar model can be used to describe the structure of IPC-7 (Figure 4 d), but this time 50 % D4R units and 50 % S4R units connect the layers. IPC-7 contains 14-ring pores, and can therefore be described as a zeolite with extra-large pores. IPC-7 is rather a defective material, and TEM images (Figure S8 and S9) show different types of defects in the material. It should be noted that both IPC-6 and IPC-7 are best described as disordered zeolites, but they are not intergrowth structures.

The continuous control over the surface area and microporosity of zeolites is particularly important in the design of new processes that are dependent on porosity. Applications based on gas adsorption are particularly sensitive to the accessible surface area, and tuning the surface area is important to ensure the right balance between diffusion and activity/selectivity. It is noteworthy that there has been much renewed interest in zeolites with small pores, as they are ideal candidates for several emerging applications, such as deNO_*x*_ catalysis for diesel engines[26] and gas-storage technologies.[27] A problem with many of the zeolites with extra-large pores prepared using traditional methods is the presence of Ge, which inevitably makes them less hydrolytically stable. Here we have shown that zeolites with extra-large pores can be prepared Ge-free, thus markedly increasing their stability toward water, and therefore opening up potentially new applications for zeolite materials with large pores. It is also important to note that similar chemistry may be possible with several other zeolite structures. We already published an ADOR-type process with a zeolite that has the IWW structure type.[28] While we cannot yet control the porosity in any other system, there seems a great likelihood that this method will be general to zeolites that have the correct structural and compositional properties.
