# Supplementary material for: Zeolites with Continuously Tuneable Porosity
Source: Angew Chem Int Ed Engl. 2014 Oct 5;53(48):13210–4. doi: 10.1002/anie.201407676 (PMC4501317; doi:10.1002/anie.201407676)
Supplement: Supplementary file 1 [file anie0053-13210-sd1.pdf]

Angewandte Chemie
Eine Zeitschrift der Gesellschaft Deutscher Chemiker

Supporting Information
© Wiley-VCH 2014
69451 Weinheim, Germany

Zeolites with Continuously Tuneable Porosity**

Paul S. Wheatley, Pavla Chlubná-Eliášová, Heather Greer, Wuzong Zhou, Valerie R. Seymour, Daniel M. Dawson, Sharon E. Ashbrook, Ana B. Pinar, Lynne B. McCusker, Maksym Opanasenko, Jiří Čejka, and Russell E. Morris**

anie_201407676_sm_miscellaneous_information.pdf

Supplementary Information

S1 Synthesis

S1.1 Synthesis of Structure Directing Agent – (6R,10S)-6,10-dimethyl-5-azoniaspiro[4,5]decane.

This procedure was similar to that found in the synthesis of IM-12.[1]. The bromide salt was then ion exchanged with Ambersep 900-OH resin. Typically, (6*R*,10*S*)-6,10-dimethyl-5-azoniaspiro[4,5]decane bromide (40 g, 161.2 mmol) was dissolved in distilled water (110 mL) to which was added Ambersep 900-OH (40 g) and stirred for 16 hours. This was then filtered and washed with distilled water (10 mL). This was repeated 4 times in total so that approximately a 0.6M solution of (6*R*,10*S*)-6,10-dimethyl-5-azoniaspiro[4,5]decane hydroxide was obtained.

S1.2 Synthesis of germanium containing UTL (Ge-UTL).

A gel with the following molar composition 0.8 SiO_2: 0.4 GeO_2: 0.36-0.4 ROH: 30-32 H_2O was prepared by dissolving amorphous germanium dioxide (10.80 g, 103.2 mmol, 99.999% Acros) in a 0.625M (6*R*,10*S*)-6,10-dimethyl-5-azoniaspiro[4,5]decane hydroxide solution (150 mL). Then silicon dioxide (12.46 g, 207.7 mmol, Cab-o-sil) was added portion-wise into the solution and the resulting mixture was stirred at room temperature for a further 30 minutes. The gel was then transferred into a Teflon-lined autoclave and heated at 175 °C for 20 days under static conditions or for 7 days under rotation conditions. The solids were recovered by filtration, washed with copious amounts of distilled water and air dried overnight.

Calcination of the as-synthesised zeolite was carried out to remove the SDA. Typically, the zeolite was heated to 550 °C at a rate of 1 $°Cmin^{-1}$, and held for 6 hours and cooled to room temperature at a rate of 2 $°Cmin^{-1}$ under an atmosphere of air.

S1.3 Hydrolysis of Ge-UTL.

The protocol for investigating the mechanism of the ADoR process is as follows: 1g portions of Ge-UTL were added to room temperature 250 mL aliquots of pure water and aqueous solutions of HCl at the required concentration (from 0.1 M, 6 M and 12 M) and 0.0025 M tetramethyl ammonium hydroxide, so that there were six reactions under each set of conditions. After 5 minutes at room temperature the solid from one of the reactions at each acidity was recovered. The remaining five reactions for were heated at 85/95 °C for varying amounts of time – 1, 2, 8, 16 and 24 hours respectively. The solid from each of the reactions was recovered by filtration and the solids examined by powder X-ray diffraction (PXRD) and scanning electron microscopy fitted with energy dispersive X-ray (EDX) analysis. The solution from each reaction was also recovered and analysed for its chemical composition.

For the full range of acidity from 0.1 to 12 M, further samples of calcined Ge-UTL were hydrolysed in hydrochloric acid solution (variable molarity, 0.1M – 12M) with a w/v ratio of 1 g/160 mL at 95 °C for 17-24 hours hours. The solid product was recovered by filtration, washed with copious amounts of distilled water and air dried overnight. Additional hydrolysis experiments were completed using 12M hydrochloric acid to investigate the effect of time. Hydrolysed samples were then calcined as above.

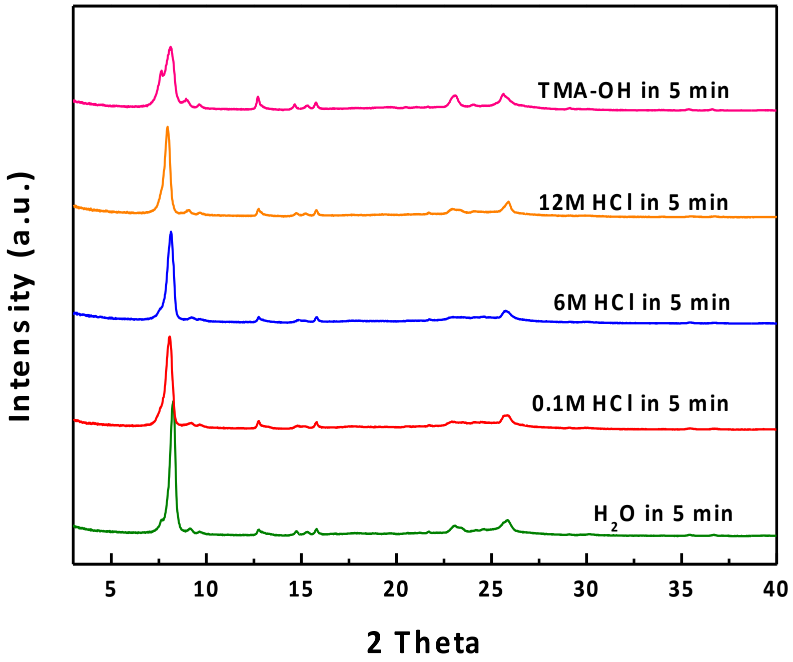

Figure S1 PXRD patterns of UTL samples that have been hydrolysed at room temperature for 5 minutes to form IPC-1P. The peak positions are not dependent on the acidity except for the TMA-OH treated sample. However, the PXRD patterns of this sample do become similar to all the others after treatment for a longer time. The measured Si/Ge ratio for these samples is in the range 25-37 and shows no trend with conditions.

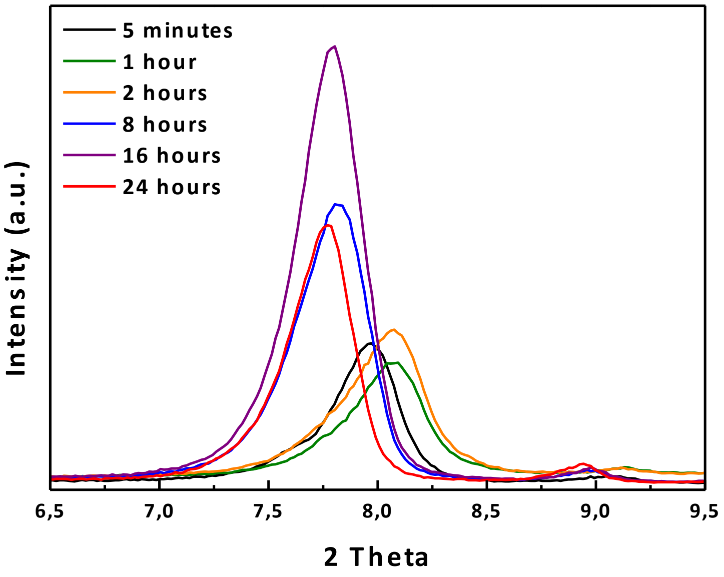

Figure S2 Evolution of the position of the 200 reflection on treatment with 12 M HCl for 5 minutes at RT and then 1, 2, 8, 16 and 24 hours at 85 °C. The final peak position is moved to smaller 2θ values, indicating an increase in the interlayer spacing.

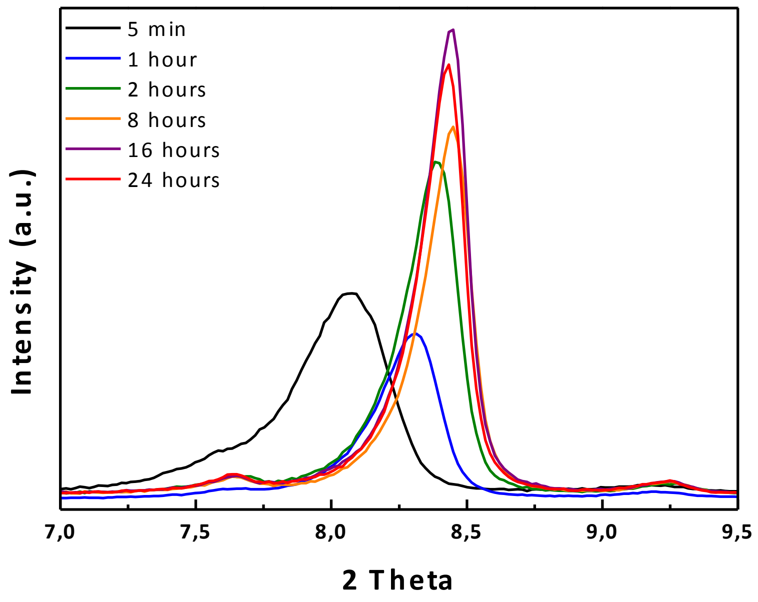

Figure S3 Evolution of the position of the (200) reflection on treatment with 0.1 M HCl for 5 minutes at RT and then 1, 2, 8, 16 and 24 hours at 85 °C. The final peak position is moved to larger 2θ values, indicating a decrease in the interlayer spacing.

S2 Characterisation of Zeolites

S2.1 Laboratory X-ray Diffraction

X-ray diffraction data were collected on a Panalytical Empyrean diffractometer using Cu Kα_1 radiation operating in reflection geometry.

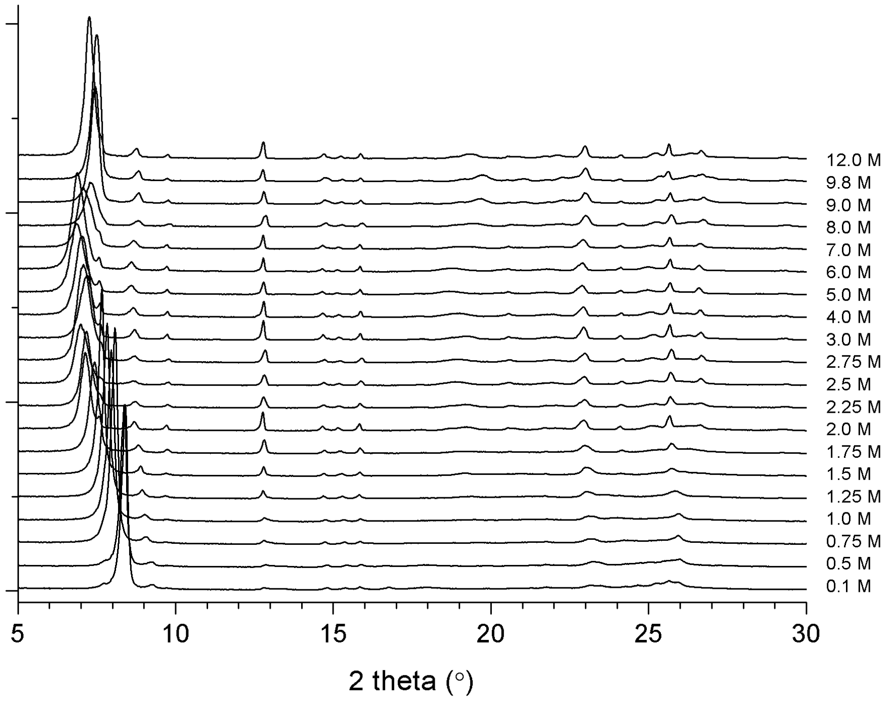

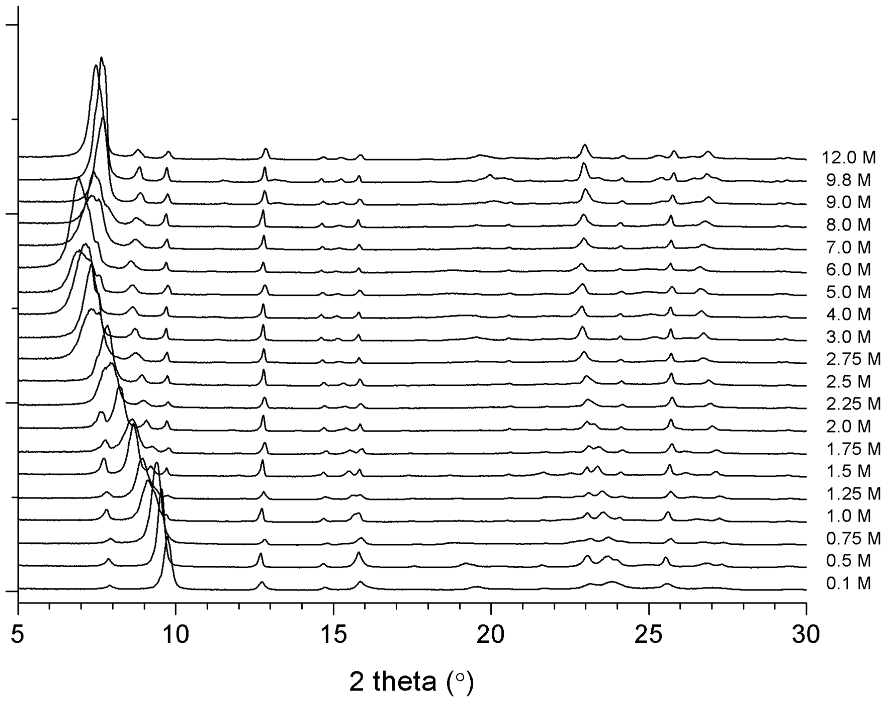

Figure S4 - PXRD patterns of Ge-UTL after hydrolysis at 95 °C using the indicated concentration of HCl, measured before (top) and after calcination of the products (bottom).

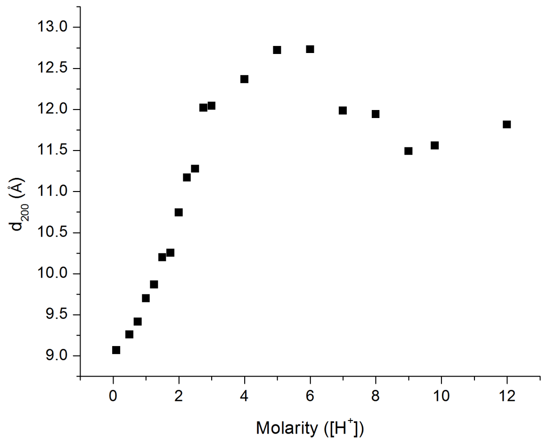

Figure S5 – Correlation of the position of the d-spacing of the 200 reflection with molarity after hydrolysis and calcination.

S2.2 Synchrotron Powder X-ray diffraction of IPC-6

Powder diffraction data were collected on the Materials Science Beamline at the Swiss Light Source (SLS) in Villigen, Switzerland[1] (Table S1). Rietveld refinement was performed using the XRS−82 suite of programmes,[2] and the structure drawings were produced using CrystalMaker.[3] The profile plots were prepared with the programme ppp14.[4]

The diffraction pattern of IPC-6 was indexed with the programme TREOR[5] implemented in the software CMPR[6] on a monoclinic unit cell with parameters a = 20.78, b = 13.89, c = 12.30 Å, b = 101.48º. The systematic absences were indicative of

body centring (space groups *I1*, *I2* or *I2/m*). This unit cell was transformed to the equivalent *C*-centred one (*a* = 21.94 Å, *b* = 13.89 Å, *c* = 12.30 Å, *b* = 112º) to facilitate comparison with the **UTL**-type framework[7], from which it had been prepared, and the other **UTL**-related frameworks (**PCR** and **OKO**). The *b* and *c* parameters are similar to those of **UTL**, which is consistent with the **UTL** layers remaining intact after the disassembly and reassembly process. A Le Bail fit (no structural model) of the diffraction pattern was performed in the space group *C2/m*, and the zero correction, sample displacement, half widths and unit cell parameters refined. The *h*00 reflections were found to be significantly broadened, so anisotropic line broadening in that direction was also refined.

From the d_{200} spacing, which is indicative of the interlayer spacing, it was thought that the framework structure of IPC-6 might be a mixture of the **OKO** and **PCR** framework types, composed of **UTL** layers stacked along *a*, connected either through a single-four ring (A) as in **OKO** or directly through an oxygen atom (B) as in **PCR**. Alternation of these two (AB) would result in a d_{200} spacing (10.18 Å) that lies between those of the **OKO** and **PCR** frameworks (11.37 Å and 9.13 Å, respectively). However, this model has a primitive unit cell. Inspection of the TEM images showed that the layers are not stacked in an ordered fashion, *i.e.* the sequence of interlayer distances observed in the electron microscopy images does not correspond to a regular (AB)(AB)(AB) sequence. The layer connections are arranged almost randomly (e.g. (BA)(AB)(BA)(AB)(AB)(BA)(AB)), but correspond to approximately 50% (AB) and 50% (BA) sequences. In other words, on average, a unit cell contains 0.5(AB) and 0.5(BA) connections. The average length of *a* does not change, because it is the same for both arrangements. However, the stacking disorder does cause the reflections along the *h*00 direction to broaden.

A model for the average framework structure was built by superimposing two structures: one with the (AB) connection sequence (*P2/m*) and the second with the same structure shifted by (1/2,1/2,0) to give a (BA) connection sequence (also *P2/m*). This model is *C*-centred, as the indexing indicated, and describes a 50:50 mixture of the two connections. The occupancy parameters for all framework atoms were set to 0.5. With this relatively simple model, the positions of the superimposed layers allow either connection to occur between layers, and in this way a switch from (AB) to (BA) stacking can occur. However, the model implies that there are no extended connections of just one type. Geometric restraints were imposed on the bond distances and angles of the framework atoms corresponding to one of the two superimposed structures, and the second was constrained to retain a strict (1/2,1/2,0) shift. Neutral scattering factors were employed for all atoms. The crystallographic data are given in Table S2, the atomic parameters in Table S3 and the profile fit for this model is shown in Figure S3. The bond distances and angles are reasonable for an all-silica framework (Si−O distances between 1.58 Å and 1.63 Å, Si−O−Si angles between 133.8º and 177.4º and O−Si−O angles between 105.6º and 111.5º). The difference electron density map calculated for the final structure was featureless, indicating that the electron density is well-described by the model. However, there are still some significant differences apparent in the profile plot (Figure S3). This was first thought to be an effect of a preferred orientation of the thin plate-like crystallites during the data collection, but no clear direction for the differences could be established. Therefore, it is assumed that the differences result from the fact that the diffraction data is complicated by a significant amount stacking defects. A more rigorous simulation of the stacking disorder is planned.

Table S1. X–ray powder diffraction data collection parameters for the samples IPC-6.

Synchrotron facility	SLS
Beamline	Material Science
Diffraction geometry	Debye-Scherrer
Detector	Mythen II Si microstrip
Monochromator	Si 111
Wavelength	0.7086 Å
Sample	Rotating 0.3 mm capillary
Nominal step size	0.004 °2θ
Detector positions	4
Time per pattern	20 s
2θ range	1.0–120°2θ (only 2.0–37.3 °2θ was used in the refinement)

Table S2. Crystallographic data from the Rietveld refinement of IPC-6.[a]

Chemical composition	$[Si_{64}O_{128}]$	
Unit cell		
a		21.9379(9) Å
b		13.8895(2) Å
c		12.2991(3) Å
β		112(3)°
Space Group		*C2/m*
Standard peak (*hkl*, 2θ)		*0 2 0*, 5.85°
Peak range (FWHM)		15
Data points		7861
Contributing reflections		842
Geometric restraints		218
Si–O	1.61(1) Å	74
O–Si–O	109.5(20)°	106
Si–O–Si	145(8) °	38
Parameters		
structural		154
profile		11
R_F		0.151
R_{wp}		0.183
R_{exp}		0.146

[a] The numbers given in parentheses are the esd's in the units of the least significant digit given. Each restraint was given a weight equivalent to the reciprocal of its esd.

Table S3. Atomic parameters for the sample IPC-6[a].

Atom	x	y	z
Si1	0.4867(3)	0.6136(3)	0.1208(4)
Si2	0.6206(3)	0.6937(3)	0.2851(8)
Si3	0.7022(3)	0.7914(3)	0.5142(6)

Si4	0.7562(5)	0.0	0.4989(8)
Si5	0.7210(3)	0.7909(3)	0.7755(8)
Si6	0.7596(6)	0.0	0.8616(11)
Si7	0.8473(5)	0.0	0.1125(8)
Si8	0.9092(3)	0.7982(3)	0.1543(7)
Si9	0.8650(3)	0.7055(2)	0.9171(10)
Si10	0.8231(5)	0.5	0.8401(10)
Si11	0.7406(4)	0.5	0.9901(8)
Si12	0.7037(4)	0.5	0.3346(10)
Si13	0.8437(3)	0.7083(3)	0.3091(6)
Si14	0.8413(4)	0.7057(2)	0.5483(9)
Si15	0.8108(4)	0.5	0.5776(7)
Si16	0.7023(3)	0.7924(3)	0.1659(7)
Si17	0.6589(3)	0.6898(4)	0.9334(9)
Si18	0.7623(5)	0.0	0.2517(8)
Si19	0.1306(5)	0.0	0.2459(7)
Si20	0.0628(3)	0.7986(2)	0.2124(11)
Si21	0.8226(3)	0.5	0.2464(11)
O1	0.5	0.6264(17)	0.0
O2	0.4849(3)	0.5	0.1498(17)
O3	0.5435(5)	0.6689(7)	0.2263(6)
O4	0.6416(4)	0.7645(6)	0.2027(14)
O5	0.6593(4)	0.5944(2)	0.2981(11)
O6	0.6386(4)	0.7457(7)	0.4111(9)
O7	0.7665(5)	0.7334(6)	0.5221(9)
O8	0.6931(6)	0.7807(7)	0.6362(8)
O9	0.7119(6)	0.9044(2)	0.4911(10)
O10	0.7795(3)	0.0	0.3900(9)
O11	0.8209(7)	0.0	0.6181(9)
O12	0.6760(5)	0.7245(5)	0.8235(10)
O13	0.7968(4)	0.7538(7)	0.8300(10)
O14	0.7169(6)	0.9036(2)	0.8084(11)
O15	0.8256(7)	0.0	0.8335(13)
O16	0.7803(6)	0.0	1.0003(11)
O17	0.8889(6)	0.9059(2)	0.1094(12)
O18	0.8292(6)	0.0	0.2270(11)
O19	0.8742(6)	0.7235(5)	0.0506(8)
O20	0.9876(3)	0.7874(9)	0.1992(11)
O21	0.9259(4)	0.7496(6)	0.8945(8)
O22	0.8656(5)	0.5934(2)	0.8935(10)
O23	0.7610(5)	0.5	0.8780(10)
O24	0.6989(4)	0.5943(2)	0.9890(11)
O25	0.8050(4)	0.5	0.1076(11)
O26	0.7408(6)	0.5	0.4734(11)
O27	0.7559(5)	0.5	0.2714(14)
O28	0.1104(5)	0.7543(5)	0.3335(7)

O29	0.8638(4)	0.5961(2)	0.3031(11)
O30	0.7658(4)	0.7253(6)	0.2351(9)
O31	0.8607(5)	0.7381(5)	0.4419(8)
O32	0.8510(5)	0.5924(2)	0.5663(10)
O33	0.8002(6)	0.5	0.7001(10)
O34	0.6796(6)	0.7748(5)	0.0283(7)
O35	0.7203(5)	0.9048(2)	0.1945(9)
O36	0.8857(6)	0.7762(5)	0.2592(10)
O37	0.0833(5)	0.9085(2)	0.2153(10)
O38	0.5820(3)	0.6652(8)	0.8938(8)

[a]: Numbers in parentheses are the estimated standard deviations (esd's) in the units of the least significant digit given. Each restraint was given a weight equivalent to the reciprocal of its esd. Isotropic displacement parameters (B) were set to 1 for Si and 2 for O.

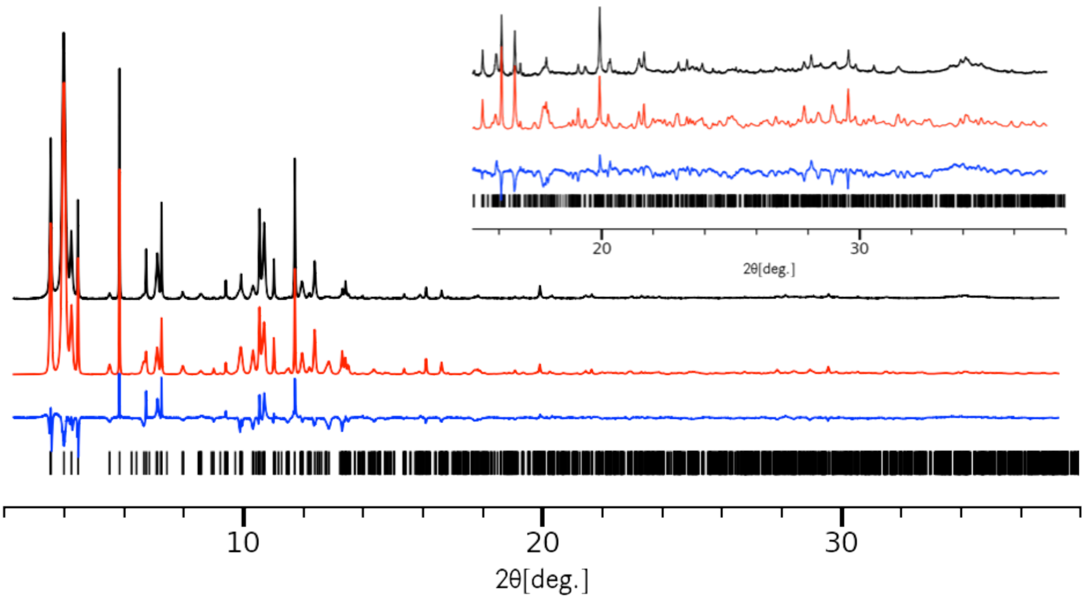

Figure S6. Observed (black), calculated (red) and difference (blue) profiles for the Rietveld refinement of IPC-6. The profiles in the inset have been scaled up by a factor of 6 to show more detail. Reflection positions are marked as vertical bars.

S2.2 Electron microscopy

The size, shape and elemental composition of zeolite crystals were examined by scanning electron microscopy (SEM) using a Jeol JSM 5600 SEM equipped with an EDX system. For the measurement, crystals were coated with a thin platinum layer by sputtering in vacuum chamber of a BAL-TEC SCD-050.

The microstructures were investigated using high resolution transmission electron microscopy (HRTEM) on a Jeol JEM-2011 electron microscope operating at an accelerating voltage of 200 kV. The Jeol JEM-2011 electron microscope is equipped with an Oxford Link ISIS SemiSTEM EDX system, which was used for confirming chemical compositions of the samples. The HRTEM images were recorded using a

Gatan 794 CCD camera. The camera length, sample position and magnification were calibrated using standard gold film methods.

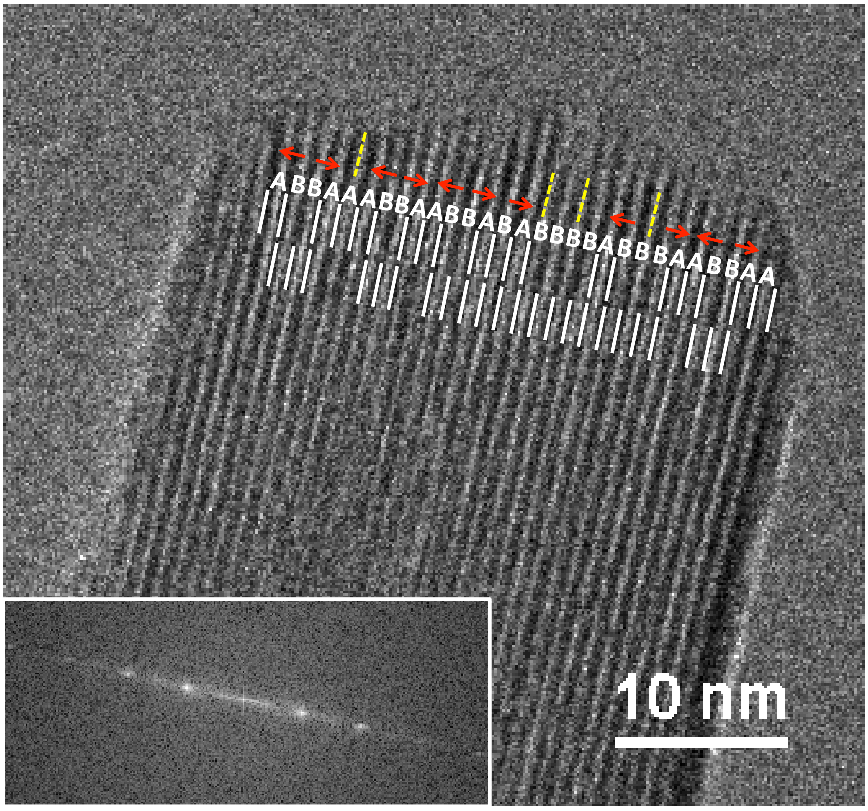

Figure S7 TEM images of particles of IPC-6 after hydrolysis using 1.5 M HCl and further calcination. Similar to the image of a different sample shown in the main paper (Figure 4), this image shows clearly the staged nature of the material with two distinct interlayer spacings of 1.1 nm (A) and 0.9 nm (B), together with occasional stacking faults (dotted yellow lines). Red arrows indicate the direction of the IPC-6 unit cell, and the inset shows the FFT of the image in 1, with the diffuse streak indicating the presence of the stacking disorder.

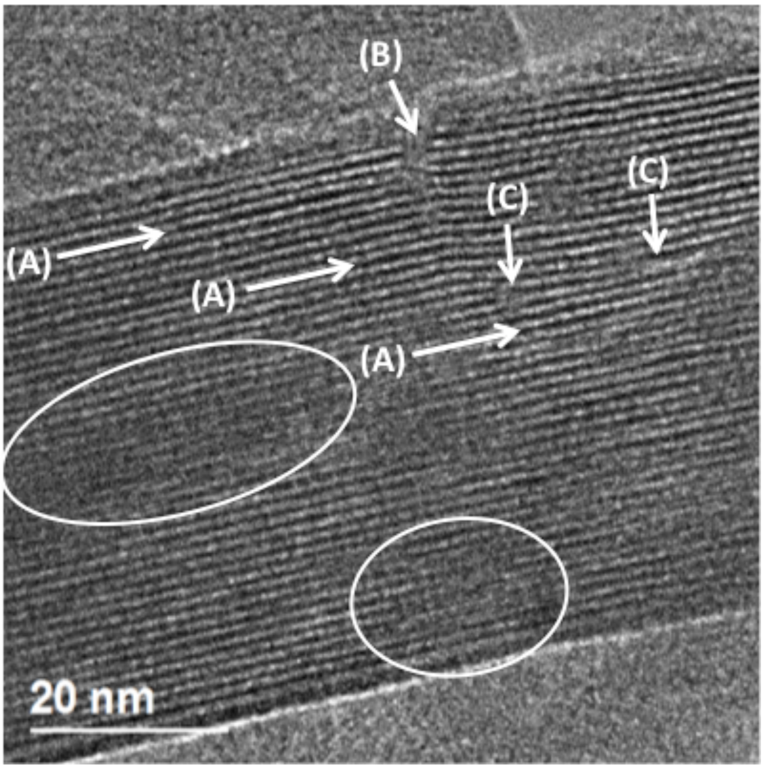

Figure S8. TEM image of the IPC-7 sample made using 5M HCl, followed by calcination. The images show very disordered materials with an average d-spacing of 1.24 nm. Local larger d-spacings (up to 1.4 nm) are detectable as marked (A). Local defects can be seen as mismatched layers (B) and where two layers merge into one, (C). Other local disordering is marked by circles.

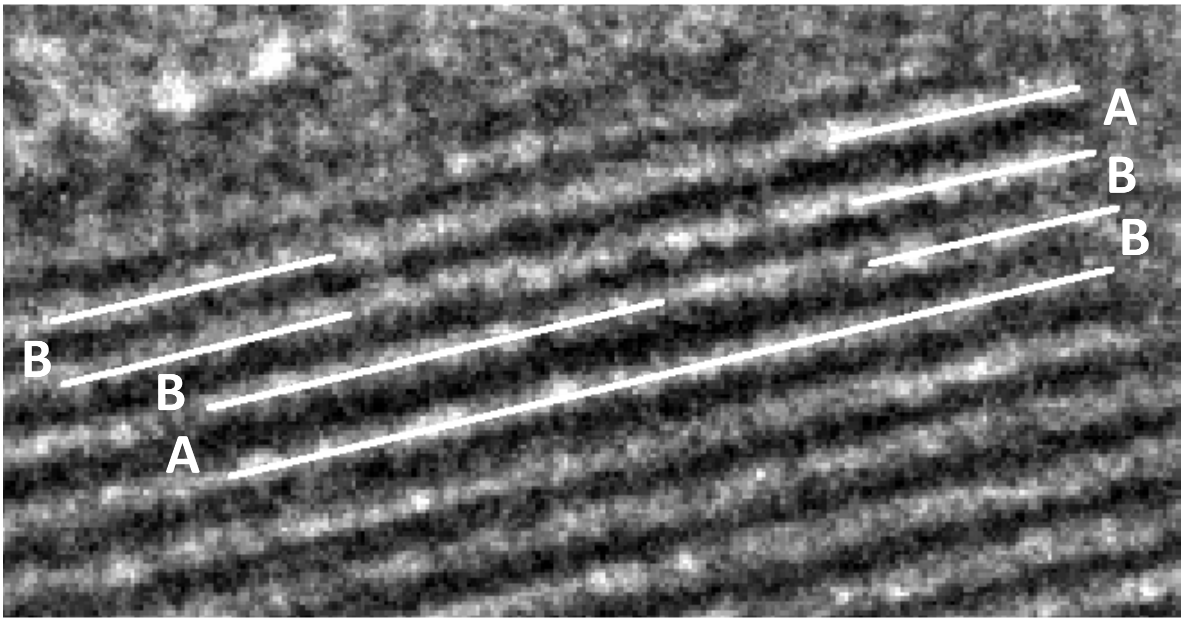

Figure S9. TEM image of the interlayer spacing in IPC-7 showing spacings of 1.4 nm (corresponding to D4R units between the layers, A) and 1.1 nm corresponding to S4R units between the layers, B. The image also shows a defect where the two different layer spacings coexist between two layers in different parts of the crystallite.

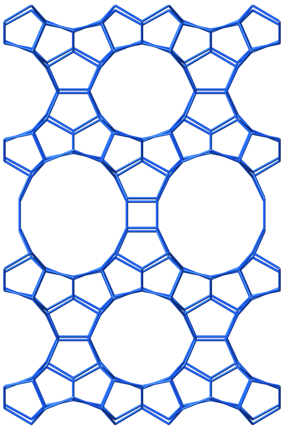

Figure S10. Proposed average structure of IPC-7 showing 50% of the layers linked by D4R units and 50% linked by S4R units. This model is consistent with the TEM image in Figure S8 and with the diffraction pattern, which gives an indexed monoclinic unit cell of a = 12.336 Å, b = 14.391 Å, c = 26.129 Å b = 99.41°
The structural model consists of a two dimensional arrangement of 14-, 12- and 10-ring channels that is consistent with the adsorption experiments.

S2.3 Nitrogen Adsorption

Nitrogen adsorption/desorption isotherms were collected at -196 °C using a Micromeritics ASAP 2020. Samples were degassed at 300 °C for 3 hours.

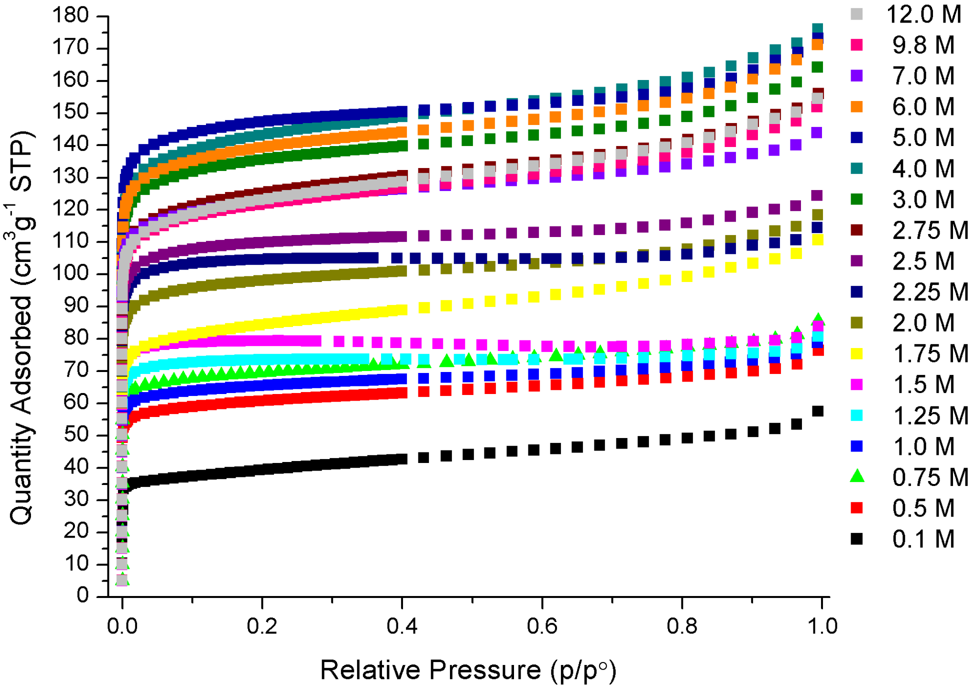

Figure S11 Adsorption isotherms for all samples after calcination.

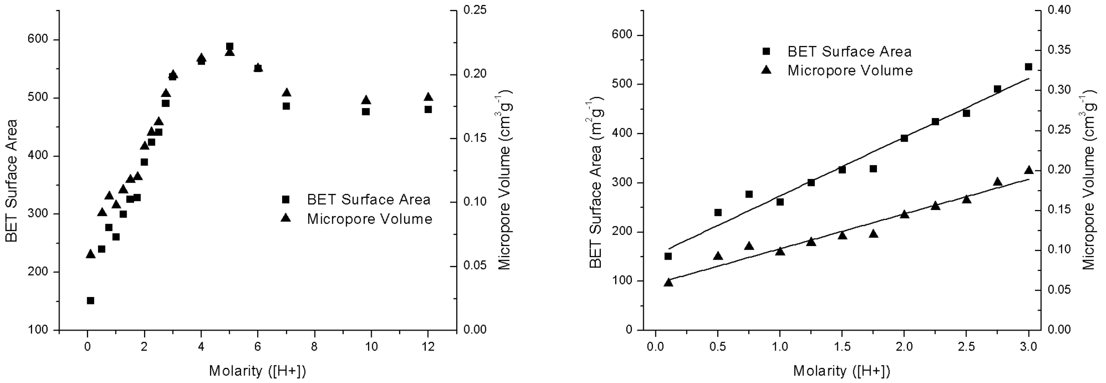

Figure S12 – Correlation of BET surface area and micropore volume with molarity of calcined samples.

S2.5 Solid-state NMR

Solid-state ^{29}Si NMR spectra were recorded using a Bruker Avance III spectrometer equipped with a 9.4 T wide-bore superconducting magnet. Samples were packed into 4 mm zirconia rotors and rotated at the magic angle at a rate of 10 kHz. Quantitative spectra were recorded with signal averaging for between 440 and 592 transients for the different samples with a recycle interval of 120 s. A pulse of flip angle 90° was applied, with $\omega_1/2\pi \approx 90$ kHz. Q^3 Si species were identified by cross polarisation (CP) from ^{1}H with a spin lock duration of 5 ms and high-power ($\omega_1/2\pi \approx 80$ kHz) TPPM ^{1}H decoupling during acquisition. For the spectra shown in Figure S several experiment with different spin lock durations are shown by the different coloured spectra. For the CP experiments, signal averaging was carried out for 1024 transients with a recycle interval of 4 s.

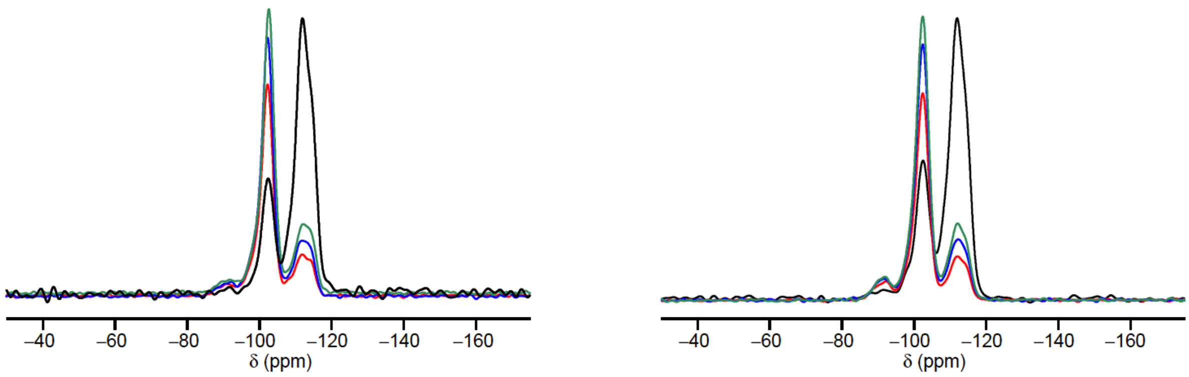

Figure S13 The direct (black) and {^{1}H} ^{29}Si CP spectra (red, blue and green) for hydrolysed Ge-UTL sample recovered after 5 minutes room temperature hydrolysis with 0.1 M HCl (left) and 12 M HCl (right).

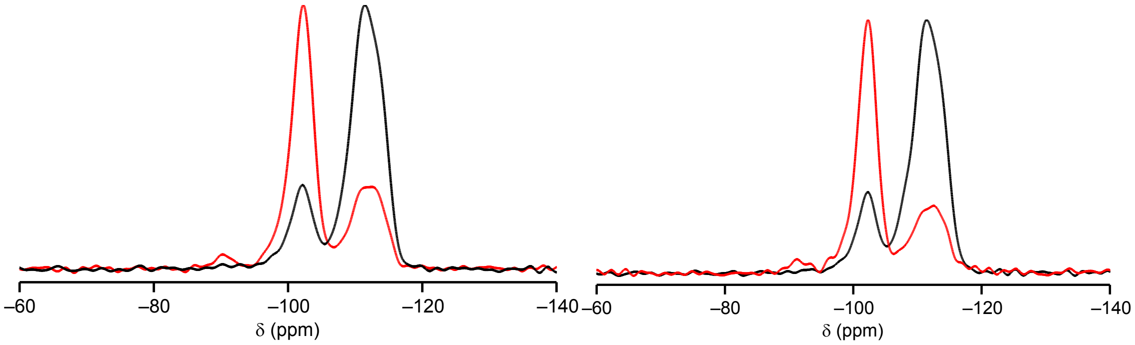

Figure S14 The quantitative MAS ^{29}Si (black) and {^{1}H} ^{29}Si CP spectra (red) spectra for samples hydrolysed at 1.5 M (left) and 6 M (right) after 17 hours at 95 °C. The Q3:Q4 ratios are 25.0:75.0 and 23.8:76.2 respectively for the 1.5 M and 6 M samples respectively, indicating the quite similar nature of the lamellar intermediates Spectra have been normalised, since the absolute intensities of the MAS spectra vary depending on number of transients averaged.

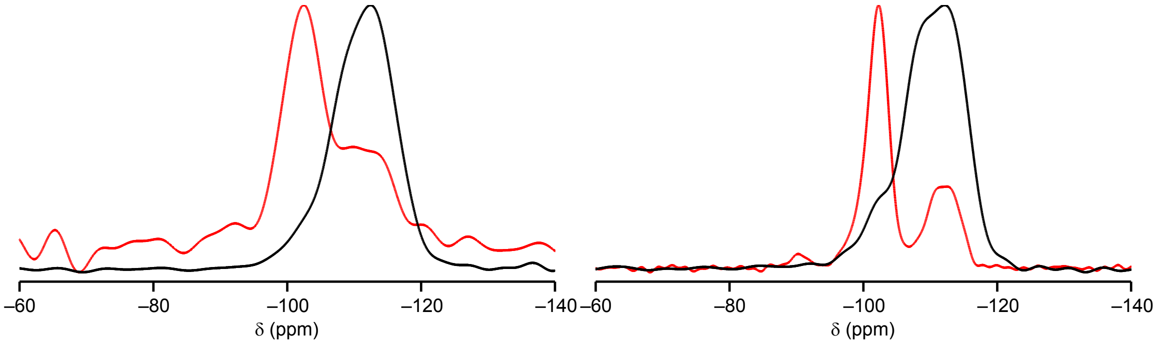

Figure S15 The quantitative MAS ^{29}Si (black) and {^{1}H} ^{29}Si CP spectra (red) spectra for samples hydrolysed at 1.5 M (left) and 6 M (right) after 17 hours at 95 °C and then calcined at 550 °C. The Q3:Q4 ratios are 5.6:94.4 and 14.7: 85.2 for the 1.5 M and 6 M samples respectively. The absolute intensities of the CP MAS spectra varied quite significantly, with the 1.5 M (IPC-6) sample showing very little signal (as evidenced by the higher noise level of the baseline). This is consistent, together with the low amount of Q3 in this sample, of a material where the interlayer connections are well

established, unlike the much more disordered 6 M sample having much more Q3 and much less well developed connections.

[1] Paillaud, J.-L., Harbuzaru, B., Patarin, J. and Bats, N.
Science, 304, 990-992 (2004)

```
data_PS114e
_audit_creation_method        'generated by CrystalMaker 8.5.4b2'
_cell_length_a               21.9379(9)
_cell_length_b               13.8895(2)
_cell_length_c               12.2991(3)
_cell_angle_alpha            90.0000(0)
_cell_angle_beta             112(3)
_cell_angle_gamma            90.0000(0)

_symmetry_space_group_name_H-M    'C 2/m'
_symmetry_Int_Tables_number       12
_symmetry_cell_setting            monoclinic
loop_
_symmetry_equiv_pos_as_xyz
'+x,+y,+z'
'1/2+x,1/2+y,+z'
'-x,+y,-z'
'1/2-x,1/2+y,-z'
'-x,-y,-z'
'1/2-x,1/2-y,-z'
'+x,-y,+z'
'1/2+x,1/2-y,+z'

loop_
_atom_site_label
_atom_site_type_symbol
_atom_site_occupancy
_atom_site_fract_x
_atom_site_fract_y
_atom_site_fract_z

  Si1   Si  0.5  0.4867(3)  0.6136(3)  0.1208(4)
  Si2   Si  0.5  0.6206(3)  0.6937(3)  0.2851(8)
  Si3   Si  0.5  0.7022(3)  0.7914(3)  0.5142(6)
  Si4   Si  0.5  0.7562(5)  0.0       0.4989(8)
  Si5   Si  0.5  0.7210(3)  0.7909(3)  0.7755(8)
  Si6   Si  0.5  0.7596(6)  0.0       0.8616(11)
  Si7   Si  0.5  0.8473(5)  0.0       0.1125(8)
  Si8   Si  0.5  0.9092(3)  0.7982(3)  0.1543(7)
  Si9   Si  0.5  0.8650(3)  0.7055(2)  0.9171(10)
  Si10  Si  0.5  0.8231(5)  0.5       0.8401(10)
  Si11  Si  0.5  0.7406(4)  0.5       0.9901(8)
  Si12  Si  0.5  0.7037(4)  0.5       0.3346(10)
  Si13  Si  0.5  0.8437(3)  0.7083(3)  0.3091(6)
  Si14  Si  0.5  0.8413(4)  0.7057(2)  0.5483(9)
  Si15  Si  0.5  0.8108(4)  0.5       0.5776(7)
  Si16  Si  0.5  0.7023(3)  0.7924(3)  0.1659(7)
```

Si17	Si	0.5	0.6589(3)	0.6898(4)	0.9334(9)
Si18	Si	0.5	0.7623(5)	0.0	0.2517(8)
Si19	Si	0.5	0.1306(5)	0.0	0.2459(7)
Si20	Si	0.5	0.0628(3)	0.7986(2)	0.2124(11)
Si21	Si	0.5	0.8226(3)	0.5	0.2464(11)
O1	O	0.5	0.5	0.6264(17)	0.0
O2	O	0.5	0.4849(3)	0.5	0.1498(17)
O3	O	0.5	0.5435(5)	0.6689(7)	0.2263(6)
O4	O	0.5	0.6416(4)	0.7645(6)	0.2027(14)
O5	O	0.5	0.6593(4)	0.5944(2)	0.2981(11)
O6	O	0.5	0.6386(4)	0.7457(7)	0.4111(9)
O7	O	0.5	0.7665(5)	0.7334(6)	0.5221(9)
O8	O	0.5	0.6931(6)	0.7807(7)	0.6362(8)
O9	O	0.5	0.7119(6)	0.9044(2)	0.4911(10)
O10	O	0.5	0.7795(3)	0.0	0.3900(9)
O11	O	0.5	0.8209(7)	0.0	0.6181(9)
O12	O	0.5	0.6760(5)	0.7245(5)	0.8235(10)
O13	O	0.5	0.7968(4)	0.7538(7)	0.8300(10)
O14	O	0.5	0.7169(6)	0.9036(2)	0.8084(11)
O15	O	0.5	0.8256(7)	0.0	0.8335(13)
O16	O	0.5	0.7803(6)	0.0	1.0003(11)
O17	O	0.5	0.8889(6)	0.9059(2)	0.1094(12)
O18	O	0.5	0.8292(6)	0.0	0.2270(11)
O19	O	0.5	0.8742(6)	0.7235(5)	0.0506(8)
O20	O	0.5	0.9876(3)	0.7874(9)	0.1992(11)
O21	O	0.5	0.9259(4)	0.7496(6)	0.8945(8)
O22	O	0.5	0.8656(5)	0.5934(2)	0.8935(10)
O23	O	0.5	0.7610(5)	0.5	0.8780(10)
O24	O	0.5	0.6989(4)	0.5943(2)	0.9890(11)
O25	O	0.5	0.8050(4)	0.5	0.1076(11)
O26	O	0.5	0.7408(6)	0.5	0.4734(11)
O27	O	0.5	0.7559(5)	0.5	0.2714(14)
O28	O	0.5	0.1104(5)	0.7543(5)	0.3335(7)
O29	O	0.5	0.8638(4)	0.5961(2)	0.3031(11)
O30	O	0.5	0.7658(4)	0.7253(6)	0.2351(9)
O31	O	0.5	0.8607(5)	0.7381(5)	0.4419(8)
O32	O	0.5	0.8510(5)	0.5924(2)	0.5663(10)
O33	O	0.5	0.8002(6)	0.5	0.7001(10)
O34	O	0.5	0.6796(6)	0.7748(5)	0.0283(7)
O35	O	0.5	0.7203(5)	0.9048(2)	0.1945(9)
O36	O	0.5	0.8857(6)	0.7762(5)	0.2592(10)
O37	O	0.5	0.0833(5)	0.9085(2)	0.2153(10)
O38	O	0.5	0.5820(3)	0.6652(8)	0.8938(8)
